# Simultaneous quantification and antiatherosclerosis effect of the traditional Korean medicine, Hwangryunhaedok-tang

**DOI:** 10.1186/s12906-015-0632-5

**Published:** 2015-04-08

**Authors:** Chang-Seob Seo, Ohn Soon Kim, Jung-Hoon Kim, Hyeun-Kyoo Shin

**Affiliations:** Herbal Medicine Formulation Research Group, Korea Institute of Oriental Medicine, 1672 Yuseong-daero Yuseong-gu, Daejeon, 305-811 South Korea; Division of Pharmacology, School of Korean Medicine, Pusan National University, Yangsan, Gyeongnam, 626-870 Republic of Korea

**Keywords:** Simultaneous quantification, Hwangryunhaedok-tang, Antiatherosclerosis effect, HPLC–PDA, Traditional Korean medicine

## Abstract

**Background:**

Hwangryunhaedok-tang (HHT) is a traditional herbal medicine that is used for the treatment of fever, inflammation, gastritis, and hypertension. In this study, we performed simultaneous determination of the five components, geniposide (**1**), baicalin (**2**), coptisine (**3**), palmatine (**4**), and berberine (**5**) in HHT by using a high-performance liquid chromatography–photodiode array (HPLC–PDA) analysis. We also evaluated the antioxidative activity of HHT and compounds **1**–**5** by measuring their effects on low-density lipoprotein (LDL) oxidation and antiproliferative abilities in vascular smooth muscle cells (VSMCs).

**Methods:**

Five compounds were separated within 40 min by using a Gemini C_18_ column (temp. 35°C; two-component gradient elution; flow rate 1.0 mL/min; detector 240 and 277 nm). The activities of HHT and compounds **1**–**5** were tested with the radical scavengers 2,2′-azino-bis(3-ethylbenzothiazoline-6-sulfonic acid) diammonium salt and 2,2-diphenyl-1-picrylhydrazyl, in thiobarbituric acid reactive substance assays, and in relative electrophoretic mobility assays using CuSO_4_-induced LDL oxidation systems. The antiproliferative effects of samples on platelet-derived growth factor (PDGF)-induced VSMC proliferation were studied by using a cell proliferation assay.

**Results:**

Regression analysis of the five major compounds showed good linearity (*r*^2^ ≥ 0.9997) in different concentration ranges. The recoveries of the five compounds were in the range 86.31–110.78%, with relative standard deviations below 2.1%; those of intra- and interday precision were 0.04–3.78% and 0.04–1.69%, respectively. HHT reduced the oxidation properties of LDL induced by CuSO_4_ and inhibited cell proliferation in PDGF-treated VSMCs. Among the five components, compound **2** could effectively suppress LDL oxidation and PDGF-induced VSMC proliferation.

**Conclusions:**

The established HPLC–PDA method will help to improve quality control of HHT. The results demonstrate that HHT has antiatherosclerotic activity and that it functions by modulating LDL oxidation and VSMC proliferation. The effects of HHT may be attributed, at least I part, to compound **2**.

## Background

Traditional herbal formulas are generally composed of various herbs and have been used for the prevention of and therapy for a variety of diseases for thousands of years. Moreover, because herbal medicines tent to have a mild effect and few side effects, many people are becoming increasingly interested in traditional herbal medicines [1-4]. Hwangryunhaedok-tang (HHT) is a traditional Korean herbal medicine consisting of four medicinal herbs, Coptidis Rhizoma, Scutellariae Radix, Phellodendri Cortex, and Gardeniae Fructus in 1:1:1:1 proportions and is called Orengedokuto in Japan and Hwanglianjiedu-tang in Chinese [5]. HHT has been used clinically for the treatment of various symptoms including inflammatory diseases [6,7], gastrointestinal disorders [8], diabetes mellitus [9], brain injury [10-12], and acute liver injury [13,14]. Recently, the preventative effect of HHT on atherosclerosis was reported in models *in vivo* [15]. However, the underlying antiatherosclerotic mechanism of HHT has not yet been thoroughly elucidated. In this study, we investigated the antioxidant effects of HHT on low-density lipoprotein (LDL) and antiproliferative effect on vascular smooth muscle cells (VSMCs), which are key atherosclerotic events [16,17]. Furthermore, chromatographic analysis was performed by using a high-performance liquid chromatography–photodiode array (HPLC–PDA) system to enable the simultaneous quantification of five major compounds, geniposide (**1**) in Gardeniae Fructus, baicalin (**2**) in Scutellariae Radix, and coptisine (**3**), palmatine (**4**), and berberine (**5**) in Coptidis Rhizoma and Phellodendri Cortex, for quality control of HHT.

## Methods

### Plant materials

The four crude herbs that make up HHT, Coptidis Rhizoma, Scutellariae Radix, Phellodendri Cortex, and Gardeniae Fructus, were purchased from Omniherb (Yeongcheon, Korea) and HMAX (Jecheon, Korea). The origin of each herbal medicine was taxonomically confirmed by Prof. Je Hyun Lee, Dongguk University, Gyeongju, Korea. Voucher specimens (2008–KE20–1 through KE20–4) have been deposited at the Herbal Medicine Formulation Research Group, Korea Institute of Oriental Medicine.

### Chemicals and reagents

**Figure 1:**
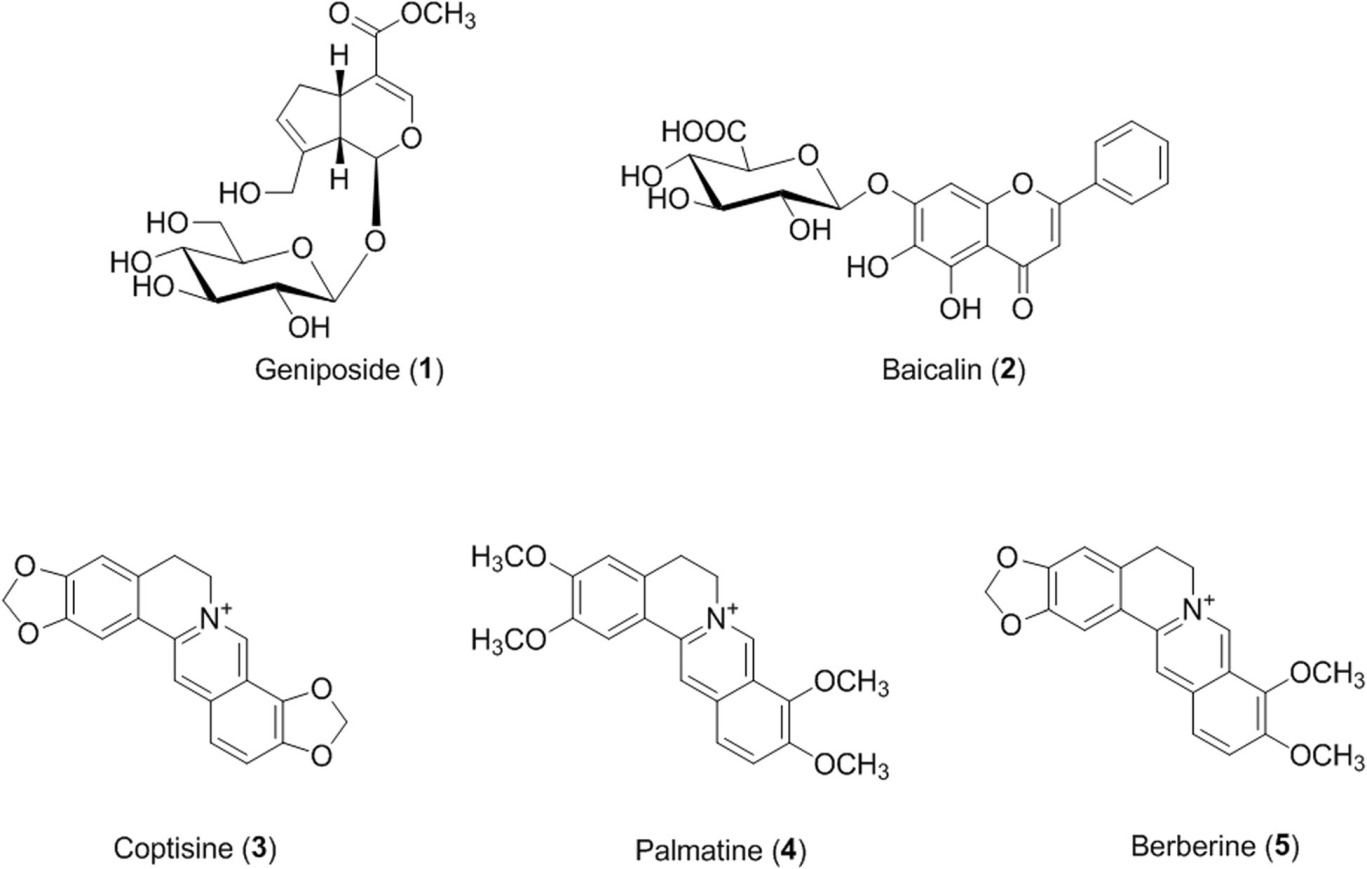
**Chemical structures of the compounds 1–5 found in HHT.**

Compounds **1**–**5** (all purity ≥ 98.0%, Figure 1) were purchased from Wako (Osaka, Japan). The HPLC-grade reagents methanol, acetonitrile, and water were obtained from J.T. Baker (Phillipsburg, NJ, USA). Sodium dodecyl sulfate (SDS) and phosphoric acid were obtained from MP Biomedicals (Solon, OH, USA) and Daejung Chemicals & Metals Co., Ltd (Daejeon, Korea), respectively. 2,2′-azino-bis(3-ethylbenzothiazoline-6-sulfonic acid) diammonium salt (ABTS) and 2,2-diphenyl-1-picrylhydrazyl (DPPH) were purchased from Sigma-Aldrich (St. Louis, MO, USA). LDL and VSMC were purchased from Biomedical Technologies (Stoughton, MA, USA) and American Type Culture Collection (ATCC, Manassas, VA, USA), respectively.

### Apparatus and conditions

A Shimadzu LC-20A HPLC system (Shimadzu, Kyoto, Japan) consisting of a system controller (CBM-20A), a solvent delivery unit (LC-20AT), an on-line degasser (DGU-20A_3_), a column oven (CTO-20A), a sample autoinjector (SIL-20 AC), and a photodiode array (PDA) detector (SPD-M20A). The data were processed by LCsolution software (version 1.24, Shimadzu, Kyoto, Japan). The analytical column used for the separation of the five components was a Phenomenex Gemini C_18_ (250 × 4.6 mm; particle size 5 μm, Torrance, CA, USA). The mobile phases consisted of solvent A (10%, v/v, acetonitrile in 0.2% SDS with phosphoric acid 200 μL/L) and solvent B (acetonitrile). The gradient conditions of the two mobile phases were: 10 → 40% B in 20 min, then 40 → 50% B in 20 min, then 50 → 100% B in 10 min, then 100 → 10% B in 5 min; the re-equilibrium time was 15 min. Column temperature was maintained at 35°C. The analysis was carried out at a flow rate of 1.0 mL/min, with PDA detection at 240 nm for iridoid and alkaloids and 277 nm for flavonoid compounds. The injection volume was 10 μL.

### Preparation of standard solutions

Each stock solution of reference compounds **1**–**5** was accurately weighed and dissolved in methanol at a concentration of 1,000 μg/mL. All the stock solutions were kept at 4°C in a refrigerator until use and diluted to the appropriate concentration range to establish calibration curves.

### Preparation of sample solutions

A decoction of HHT was prepared in our laboratory from a mixture of chopped crude herbs. HHT was prepared as described in Table 1 and extracted with distilled water at 100°C for 2 h under pressure (98 kPa) using an electric extractor (COSMOS-660; Kyungseo Machine Co., Incheon, Korea). The extract was evaporated to dryness and freeze-dried (17.1% yield). Lyophilized HHT extract (250 mg) was dissolved in distilled water (25 mL), and then the solution was passed through a 0.2 μm syringe filter (Woongki Science, Seoul, Korea) before analysis by HPLC.

**Table 1:** **Composition of HHT**

**Scientific name**	**Latin name**	**Amount (g)**	**Supplier**	**Origin**
*Coptis chinensis*	Coptidis Rhizoma	4.5	HMAX	China
*Scutellaria baicalensis*	Scutellariae Radix	4.5	HMAX	Jeongseon, Korea
*Phellodendron chinensis*	Phellodendri Cortex	4.5	HMAX	China
*Gardenia jasminoides*	Gardeniae Fructus	4.5	Omniherb	Muju, Korea
Total amount		18.0		

### Calibration curves, range, limits of detection (LODs), and of quantification (LOQs)

Each calibration curve was established by plotting peak areas versus the concentration of standard solutions. The concentration ranges were 7.81–500.00 μg/mL for compounds **1** and **2**, 1.56–50.00 μg/mL for compounds **3** and **5**, and 4.69–300.00 μg/mL for compound **4**. To assess LOD and LOQ values, stock solutions of all reference compounds were diluted with methanol. The LOD and LOQ values were determined as signal-to-noise (S/N) ratios of 3 and 10, respectively.

### Precision and accuracy

Intra- and interday precisions were determined by using a standard addition method to prepare spiked samples, employing both standards and controls. Precisions are presented as the relative standard deviation (RSD) for intra- and interday. The repeatability of the developed method was evaluated by measuring six replicates of the mixed standard solutions. The RSD values of peak areas and retention times of each compound were used to evaluate the repeatability of the developed HPLC method. The test for recovery, which was carried out to evaluate the accuracy of the methods, was performed by adding three different concentrations (low, medium, and high) of five reference standards to 200 mg of HHT sample. This test was conducted in triplicate and evaluated by using the independently prepared calibration curves.

### Determination of antioxidant activity

#### ABTS radical scavenging activity

The ABTS radical scavenging activity of the samples was determined by using the method described Re *et al.* [18] with slight modifications. Briefly, the ABTS radical cation was produced by reacting 7 mM ABTS solution with 2.45 mM potassium persulfate, then the solution was stored in the dark at room temperature for 16 h. Prior to the assay, the solution was diluted with phosphate buffer saline (PBS, pH 7.4) to an absorbance of 0.7 at 734 nm. The ABTS^•+^ solution was then added to a 96-well plate containing the test sample. After 5 min incubation, the absorbance was immediately measured at 734 nm by using a microplate reader (Benchmark Plus, Bio-Rad. Hercules, CA, USA). The extent of decolorization was calculated as the percentage reduction of absorbance. The scavenging capability of test compounds was calculated by using the equation:

$$ \mathrm{ABTS}\ \mathrm{radical}\ \mathrm{scavenging}\ \mathrm{activity}\ \left(\%\right) = \left(1\ \hbox{-}\ {\mathrm{A}}_{\mathrm{sample}}/{\mathrm{A}}_{\mathrm{control}}\right) \times 100, $$

where A_control_ is the absorbance of the negative control and A_sample_ is the absorbance of the sample. RC_50_ values (the concentration required for 50% reduction of ABTS radical) were calculated from the concentration of sample required to reduce the absorbance by 50%.

#### DPPH radical scavenging activity

Radical scavenging activity of samples was determined by using DPPH as a free radical by the method described Moreno *et al.* [19] with some modifications. Briefly, 100 μL of various concentrations of sample was added to 100 μL of DPPH solution (0.15 mM in ethanol) in a 96-well plate. After 30 min incubation in the dark at room temperature, the absorbance was measured at 517 nm. Activity of scavenging (%) was calculated by using the above formula.

### Determination of LDL oxidation

#### Oxidation of LDL by CuSO_4_

We examined the oxidation of LDL by CuSO_4_ by using a previously described method [20]. LDL samples (500 μg protein/mL, Biomedical Technologies, Stoughton, MA, USA) were prepared at 37°C in a medium containing 10 mM phosphate buffer (pH 7.4) and various concentrations of samples. After 5 min, the oxidation was initiated by the addition of CuSO_4_ (25 μM). After 6 h oxidation, lipid peroxidation and electrophoretic mobility of LDLs were measured as described below.

#### Determination of thiobarbituric acid reactive substance (TBARS)

Lipid peroxidation of LDLs was estimated by determinng the level of malondialdehyde (MDA) generated by using a TBARS assay kit (BioAssay Systems, Hayward, CA, USA) according to the manufacturer’s protocols [21]. After oxidation, 50 μg of LDLs was mixed with 200 μL of thiobarbituric acid (TBA) and incubated at 100°C for 30 min. Upon completion of the reaction, the absorbance at 535 nm was measured by using a microplate reader.

#### Relative electrophoretic mobility (REM) assay

The electrophoretic mobility of LDLs was measured by using agarose gel (0.8% agarose in TAE buffer) electrophoresis and Coomassie Brilliant Blue R-250 staining. Electrophoresis was performed at 100 V for 30 min. REM was defined as the ratio of the distances migrated from the origin by oxLDL versus native LDL [22].

### Vascular smooth muscle cell (VSMC) proliferation assay

Rat embryonic thoracic aorta smooth muscle-derived A7r5 cells were obtained from the American Type Culture Collection (ATCC, Manassas, VA, USA) and cultured as a monolayer culture at 37°C in a humidified atmosphere of 5% CO_2_, 95% air in Dulbecco’s modified Eagle’s medium (DMEM, Gibco Inc., NY, USA), containing 10% v/v fetal bovine serum (FBS, Gibco Inc., Grand Island, NY, USA) and 1% penicillin–streptomycin (P/S). Upon the attainment of 70–80% confluency, the cells were incubated in serum-free DMEM containing 0.5% bovine serum albumin for 20–24 h. Cytotoxicity and proliferation assays were performed by using the Cell Counting Kit-8 (CCK-8) as described by the manufacturer (Dojindo Laboratory, Kumamoto, Japan). Briefly, cells were seeded onto 96-well plates and grown in a final volume of 100 μL media per well. After treatment as indicated in the text for 24 h, 10 μL of kit reagent was added and the sample was incubated for an additional 3 h. Absorbance was measured at a wavelength of 450 nm by using a microplate reader.

### Statistical analysis

Statistical evaluation of the results was performed by using one-way analysis of variance (ANOVA) followed by Dunnett’s multiple comparison test by using GraphPad InStat 3.05 software (GraphPad Software Inc, San Diego, CA, USA).

## Results and discussion

### Optimization of chromatographic conditions

**Figure 2:**
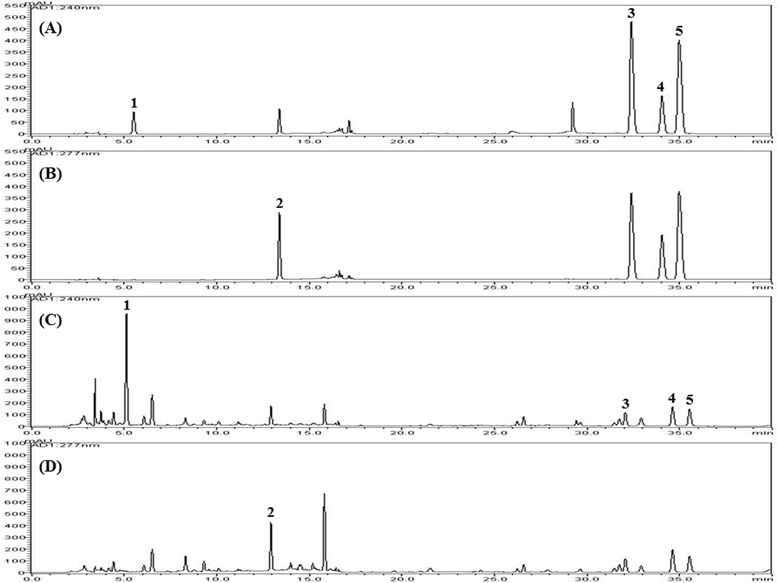
**HPLC chromatogram of the standard mixture of five compounds with detection at 240 nm (A) and 277 nm (B), HHT sample at 240 nm (C), and 277 nm (D).** Geniposide (**1**), baicalin (**2**), coptisine (**3**), palmatine (**4**), and berberine (**5**).

HPLC conditions such as column type, column temperature, and mobile phases were optimized to achieve the simultaneous separation of five analytes including one iridoid, geniposide (**1**), one flavonoid, baicalin (**2**), and three alkaloids, coptisine (**3**), palmatine (**4**), and berberine (**5**). For the separation of compounds **1**–**5**, columns types (Phenomenex Gemini C_18_, Waters SunFire C_18_, and OptimaPak C_18_ column), column temperatures (30, 35, and 40°C), and various mobile phases (acids including acetic acid and phosphoric acid and buffers such as SDS and ammonium acetate, and organic solvent with methanol and acetonitrile) were examined. By comparing the peak shape, resolution, and baselines of the target compounds under different conditions, the most satisfactory conditions were selected as Phenomenex Gemini C_18_ column (250 × 4.6 mm, 5 μm) with gradient elution of 10% v/v, acetonitrile in 0.2% SDS with phosphoric acid 200 μL/L–acetonitrile at 35°C for the separation. Quantitation was achieved by using PDA detection at 240 nm for compounds **1** and **3**–**5** and 277 nm for compound **2** based on retention time and UV spectra compared with those of the standards. By using the optimized HPLC conditions, the five analytes eluted within 40 min and afforded good specificity without interference from other components. Representative HPLC chromatograms of standards and the HHT extract are shown in Figure 2.

### Regression equation, linearity, LOD, and LOQ

The regression equations were calculated by plotting the peak area (*y*) versus concentration (*x*, μg/mL) of each compound by using serial dilutions of the stock solution. The correlation coefficients (*r*^2^) of compounds **1**–**5** were ≥ 0.9997, which showed good linearity. The LODs and LOQs of the investigated compounds **1**–**5** were in the range 0.34–0.87 and 1.12–2.89 μg/mL, respectively (Table 2). The results showed that the developed HPLC method was acceptable for the quantitative determination of compounds **1**–**5**.

**Table 2:** **Regression equation, linear range, correlation coefficient, LODs, and LOQs for marker compounds (** ***n*** **= 3)**

**Compound**	**Linear range (μg/mL)**	**Regression equation** ^***a***^	**Correlation coefficient (** ***r*** ^**2**^ **)**	**LOD** ^***b***^ **(μg/mL)**	**LOQ** ^***c***^ **(μg/mL)**
Geniposide	7.81 − 500.00	*y* = 14575.90*x* + 29400.74	0.9997	0.87	2.89
Baicalin	7.81 − 250.00	*y* = 41028.20*x* + 12271.19	0.9999	0.34	1.12
Coptisine	1.56 − 50.00	*y* = 45048.93*x* + 3766.28	0.9999	0.34	1.15
Palmatine	4.69 − 300.00	*y* = 37568.06*x* + 15349.20	0.9999	0.45	1.49
Berberine	1.56 − 50.00	*y* = 43158.92*x* + 4420.01	0.9999	0.39	1.30

^a^ *y*: peak area (mAU) of compounds; *x*: concentration (μg/mL) of compounds.

^b^LOD = 3 × signal-to-noise ratio.

^c^LOQ = 10 × signal-to-noise ratio.

### Accuracy and precision

The recovery and precision of the developed method are shown in Table 3. The recoveries of compounds **1**–**5** were in the range of 98.90–103.39% and the RSD values were less than 2.53%. The repeatability of the developed assay was evaluated based on peak responses and retention time by using the standard solution. The RSDs of peak responses and retention time for repeatability were < 0.44% and < 0.09% (data not shown), respectively, indicating that the HPLC assay showed good repeatability under the optimized conditions. The precisions of intra and interday variation of compounds **1**–**5** in HHT were less than 1.08% and 1.87%, respectively (Table 4). The results described above indicate that the established HPLC method was accurate and precise for the quantitative determination of HHT extract.

**Table 3:** **Recoveries for the assay of the five investigated compounds in HHT**

**Analytes**	**Spiked amount (μg/mL)**	**Detected amount (μg/mL)**	**Recovery** ^**a**^ **(%)**	**SD**	**RSD (%)**
Geniposide	20.00	19.33	96.67	1.85	1.92
	50.00	50.11	100.23	0.44	0.44
	100.00	100.87	100.87	0.24	0.24
Baicalin	16.00	13.98	87.35	1.45	1.66
	40.00	34.67	86.69	0.77	0.89
	80.00	69.04	86.31	0.54	0.63
Coptisine	2.00	2.07	103.74	1.02	0.98
	5.00	5.03	100.66	0.92	0.91
	10.00	10.97	109.74	0.31	0.28
Palmatine	5.00	4.98	99.67	2.05	2.05
	12.50	12.75	102.01	1.50	1.47
	25.00	26.13	104.53	0.83	0.79
Berberine	2.00	1.99	99.47	1.18	1.19
	5.00	5.44	108.76	1.82	1.67
	10.00	11.08	110.78	0.87	0.78

^a^Recovery (%) = Detected amount / Spiked amount × 100.

**Table 4:** **Precision of the analytical results (n = 5)**

**Compound**	**Spiked Conc. (μg/mL)**	**Intraday**			**Interday**		
		**Detected Conc. (μg/mL)**	**SD**	**RSD (%)**	**Detected Conc. (μg/mL)**	**SD**	**RSD (%)**
Geniposide	20.00	19.69	0.14	0.73	19.52	0.22	1.13
	50.00	49.99	0.14	0.29	49.95	0.12	0.24
	100.00	100.09	0.04	0.04	100.12	0.04	0.04
Baicalin	16.00	16.46	0.08	0.46	16.09	0.16	1.00
	40.00	40.17	0.10	0.24	40.09	0.15	0.37
	80.00	79.82	0.06	0.07	79.94	0.05	0.06
Coptisine	2.00	1.98	0.01	0.45	2.02	0.01	0.62
	5.00	4.67	0.07	1.59	4.72	0.04	0.77
	10.00	10.17	0.04	0.35	10.14	0.02	0.16
Palmatine	5.00	5.02	0.03	0.68	4.91	0.04	0.81
	12.50	12.20	0.05	0.41	12.33	0.05	0.43
	25.00	25.14	0.02	0.08	25.10	0.03	0.12
Berberine	2.00	1.90	0.07	3.78	1.89	0.03	1.69
	5.00	4.92	0.04	0.87	4.98	0.05	1.10
	10.00	10.06	0.03	0.31	10.03	0.02	0.22

### HHT sample analysis

The five compounds in HHT were well separated by using the developed HPLC method. The retention times of compounds **1**–**5** under the optimized HPLC assay were 5.363, 13.619, 31.642, 33.097, and 33.923 min, respectively. The contents of compounds **1**–**5** in HHT were 0.97–36.54 mg/g; the values are summarized in Table 5.

**Table 5:** **Amounts of the five marker compounds in the HHT sample by HPLC (** ***n*** **= 3)**

**Compound**	**Amount (mg/g)**			**Source** ^**a**^
	**Mean**	**SD (×10** ^**−1**^ **)**	**RSD (%)**	
Geniposide	36.54	0.27	0.07	GF
Baicalin	30.24	0.72	0.24	SR
Coptisine	0.97	0.02	0.23	CR, PC
Palmatine	10.34	0.47	0.46	CR, PC
Berberine	1.35	0.02	0.16	CR, PC

^a^GF: Gardeniae Fructus, SR: Scutellariae Radix, CR: Coptidis Rhizoma, PC: Phellodendri Cortex.

### Antioxidant activity of HHT and its components

**Figure 3:**
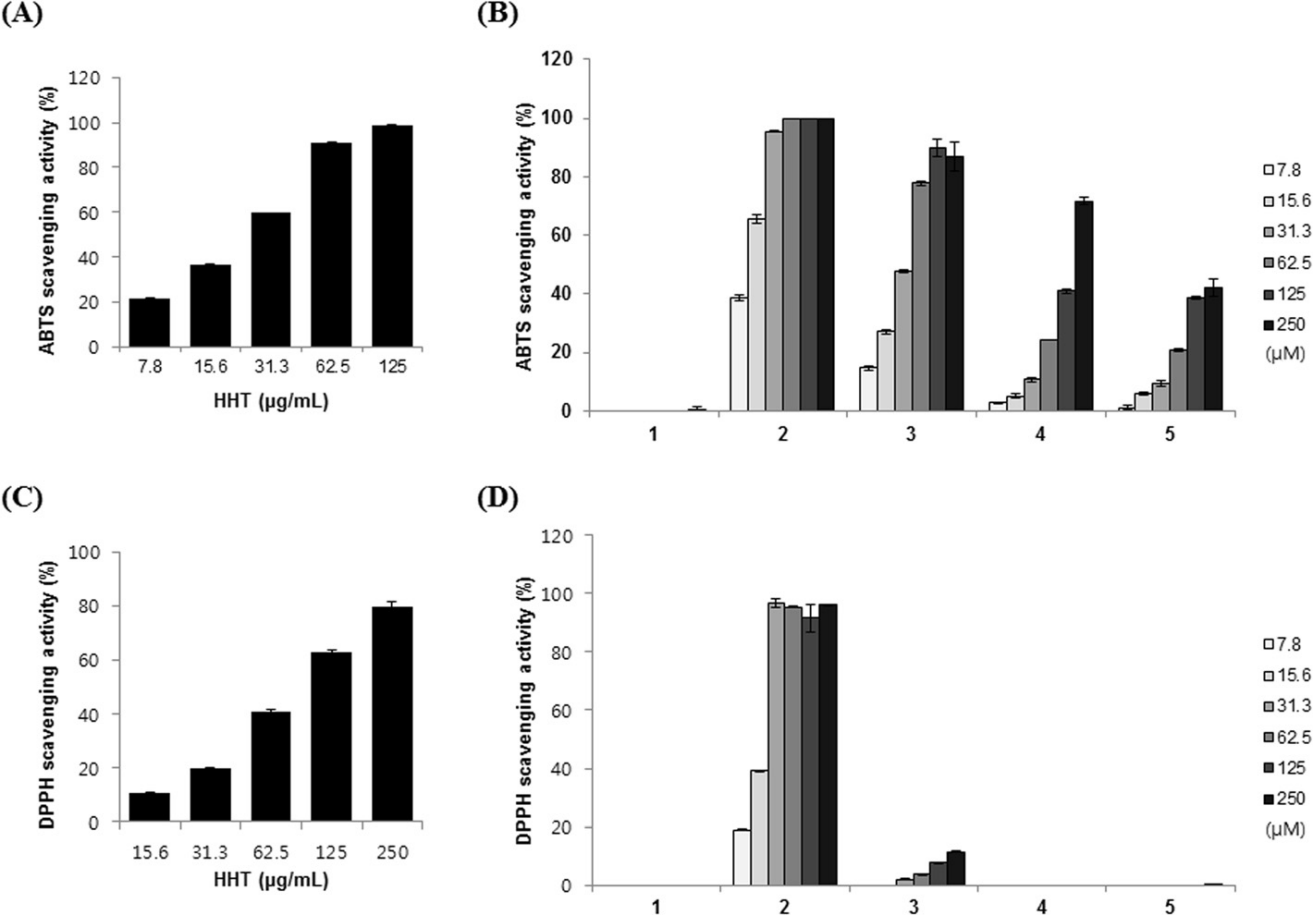
**Effects of HHT and its five components on free radical scavenging activities.** ABTS radical scavenging activity of HHT **(A)**, five components **(B)**, DPPH radical scavenging activity of HHT **(C)**, and five components **(D)**. Geniposide (**1**), baicalin (**2**), coptisine (**3**), palmatine (**4**), and berberine (**5**). The data are mean values of three experiments ± SEM (*n* = 3).

To evaluate the antioxidant activity of HHT and of compounds **1**–**5**, their respective scavenging activities on ABTS and DPPH radicals were tested. HHT showed clear antioxidant properties, which were concentration dependent (Figure 3A and C). The estimated concentration required for 50% reduction (RC_50_) against ABTS and DPPH was 25.76 ± 0.16 μg/mL and 92.97 ± 2.05 μg/mL, respectively.

Compounds **1**–**5** were also tested in ABTS and DPPH assays to determine their contribution to the antioxidant property of HHT. In the ABTS assay, compounds **2**–**4** displayed RC_50_ values of 11.28 ± 0.21, 35.72 ± 0.54, and 163.17 ± 2.64 μM, respectively (Figure 3B). The scavenging activity of compound **5** was 42% at 250 μg/mL concentration. In the DPPH assay, compound **2** had an estimated RC_50_ value of 17.76 ± 0.15 μM (Figure 3D).

### Effect of HHT and its components on Cu^2+^-mediated oxidation of LDL

**Figure 4:**
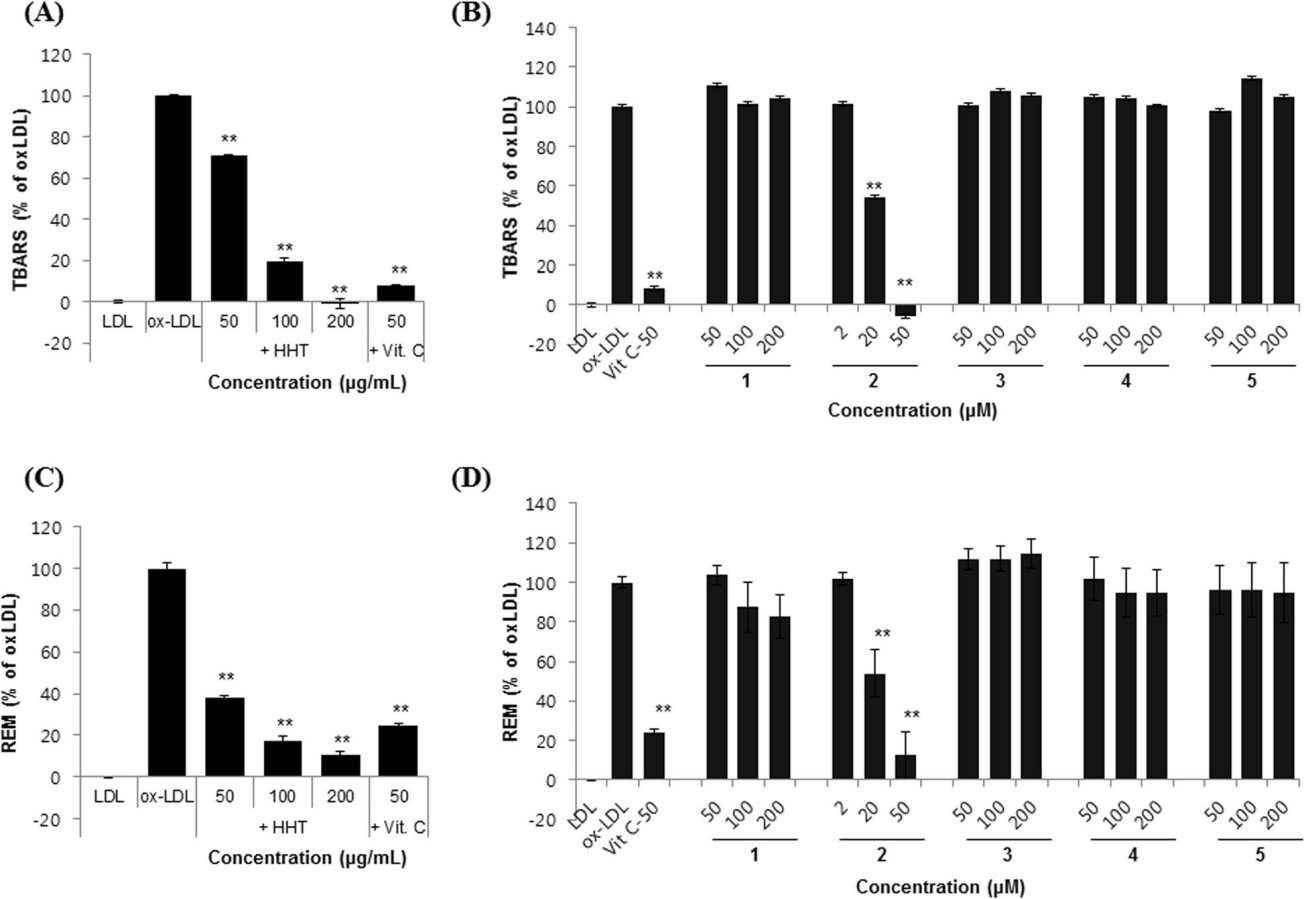
**Effects of HHT and its five components on Cu** ^**2+**^ **-induced LDL oxidation.** Indicated concentrations of samples and LDLs were incubated with CuSO_4_ for 6 h at 37°C. The TBARS levels (**A**: HHT, **B**: five components) and electrophoretic mobility (**C**: HHT, **D**: five components) of LDLs were measured by using a TBARS assay kit and agarose gel electrophoresis, respectively. Geniposide (**1**), baicalin (**2**), coptisine (**3**), palmatine (**4**), and berberine (**5**). The data are mean values of three experiments ± SEM (*n* = 3). ** *P* < 0.01 compared with the oxLDL group.

The effect of the compounds on Cu^2+^-mediated oxidation of LDL was determined by two methods. First, the degree of LDL oxidation was evaluated by the TBARS assay. Lipid peroxidation was quantified by measuring the amount of degradation by product MDA [23]. As shown in Figure 4A and B, when LDL was incubated with CuSO_4_ for 6 h, a significant increase in TBARS was detected. In contrast, HHT significantly reduced, in a concentration-dependent manner, the amount of TBARS fromed (Figure 4A). Alteration of agarose gel electrophoretic mobility reflects the increase in negative charge of LDL particles that occurred during oxidation [22]. When the oxidation was carried out in the presence of HHT, the increased electrophoretic mobility of oxidized LDL was significantly reduced (Figure 4C). As shown in Figure 4B and D, compound **2** only reduced the Cu^2+^-induced LDL oxidation.

### Effect of HHT and its components on PDGF-induced VSMC proliferation

**Figure 5:**
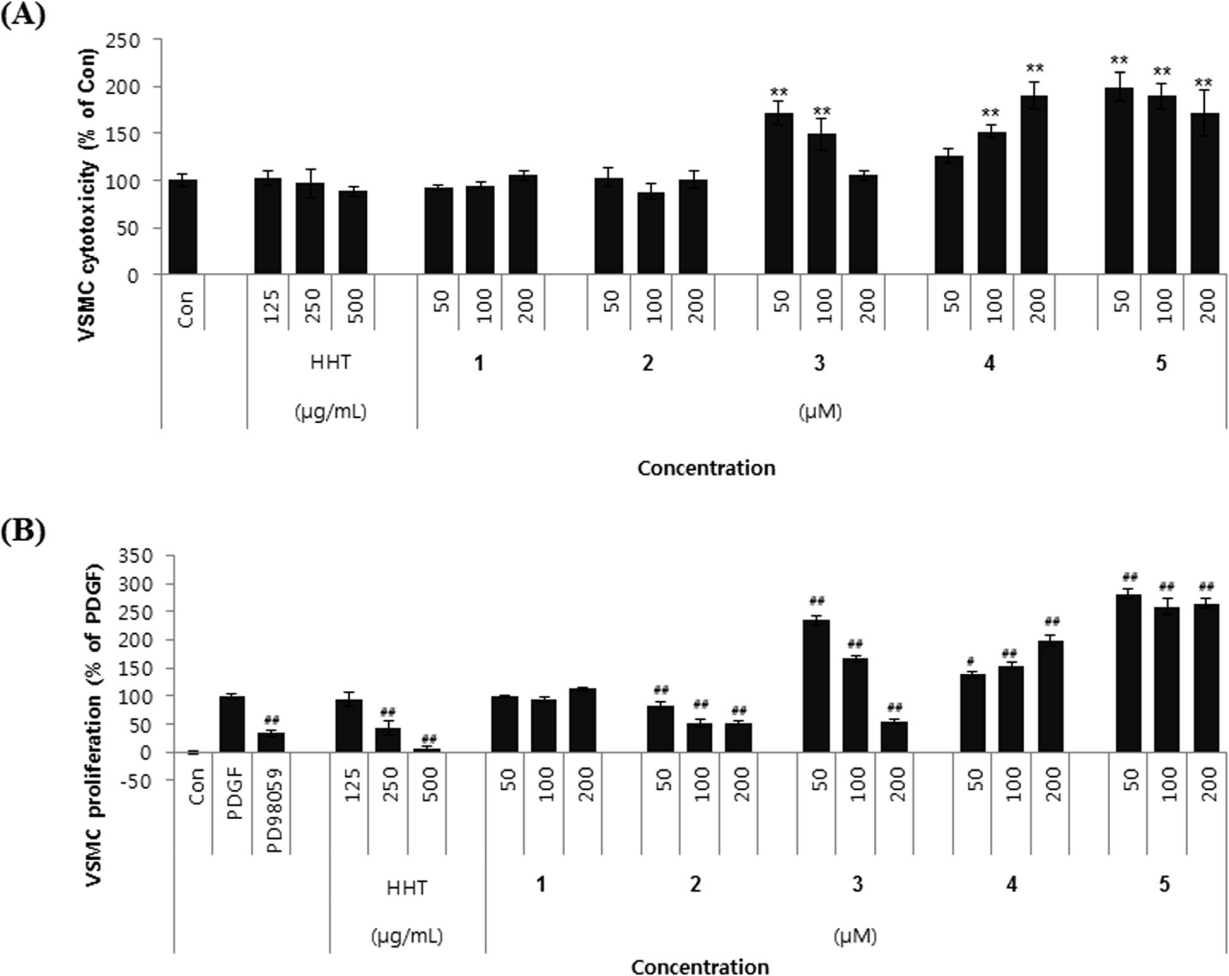
**Effects of HHT and its five components on PDGF-induced VSMC proliferation. (A)** Cytotoxicity of HHT and its five components in VSMCs. Cells were incubated with the indicated concentrations for 24 h. Cell viability was determined by using the CCK-8 assay. **(B)** Antiproliferative effects of HHT and its five components in PDGF-treated VSMCs. Quiescent VSMCs were stimulated with PDGF-BB (10 ng/mL) in the presence of the indicated concentrations of samples for 24 h and the proliferation was examined by using the CCK-8 assay. Geniposide (**1**), baicalin (**2**), coptisine (**3**), palmatine (**4**), and berberine (**5**). The data are mean values of three experiments ± SEM (*n* = 3). ^**^ *P* < 0.01 compared with the control group, ^#^ *P* < 0.05, ^##^ *P* < 0.01 compared with the PDGF group.

To determine whether HHT and its five components had any effect on cell viability, CCK-8 assays were performed on cultured rat VSMCs treated with various concentrations of samples for 24 h. As shown in Figure 5A, HHT and compounds **1** and **2** had no significant effect on the viability of cells under the experimental conditions, whereas compounds **3**–**5** induced cell proliferation. VSMCs were pretreated with different concentrations of HHT (125–500 μg/mL) and compounds **1**–**5** (50–200 μM) followed by stimulation with PDGF-BB (10 ng/mL) for 24 h. HHT and compound **2** inhibited PDGF-BB-induced proliferation of VSMCs in a concentration-dependent manner (Figure 5B). The proliferative effects of compounds **3**–**5** on PDGF-treated VSMCs were accomplished by themselves. These observations suggest that the inhibitory effect of HHT on PDGF-induced VSMC proliferation was partly attributed to compound **2**.

## Conclusions

A simple, reliable, and accurate HPLC–PDA method was developed and validated for simultaneous separation and determination of compounds **1**–**5** in the traditional Korean herbal medicine, HHT. The developed method showed good linearity, precision, and accuracy and is therefore a suitable method with which to assess the quality of HHT and its components for quality control purposes. In this study, we have shown that HHT can reduce the oxidation of LDL and inhibit PDGF-induced VSMC proliferation, which are key atherosclerotic events. Compound **2**, as one of the components in HHT, also exhibits an antioxidant effect on LDL and an antiproliferative effect on VSMCs. Although further studies are needed, these observations suggest that HHT acts, to inhibit LDL oxidation and suppress PDGF-induced VSMC proliferation, at least in part, through the effect of compound **2**.

## References

[1] Normile D (2003). Asian medicine: the new face of traditional Chinese medicine. Science.

[2] Xue T, Roy R (2003). Studying traditional Chinese medicine. Science.

[3] Jiang WY (2005). Therapeutic wisdom in traditional Chinese medicine: a perspective from modern science. Trends Pharmacol Sci.

[4] Liu S, Yi LZ, Liang YZ (2008). Traditional Chinese medicine and separation science. J Sep Sci.

[5] Hur J (2007). Donguibogam.

[6] Lu J, Wang JS, Kong LY (2011). Anti-inflammatory effects of Huang-Lian-Jie-Du decoction, its two fractions and four typical compounds. J Ethnopharmacol.

[7] Yue R, Zhao L, Hu Y, Jiang P, Wang S, Xiang L (2013). Rapid-resolution liquid chromatography TOF-MS for urine metabolomics analysis of collagen-induced arthritis in rat and its applications. J Ethnopharmacol.

[8] Ohta Y, Kobayashi T, Nishida K, Sasaki E, Ishiguro I (1999). Preventive effect of Oren-gedoku-to (Huanglian-Jie-Du-Tang) extract on the development of stress-induced acute gastric mucosal lesions in rats. J Ethnopharmacol.

[9] Yu YL, Lu SS, Yu S, Liu YC, Wang P, Xie L (2009). Huang-lian-jie-du-decoction modulates glucagon-like peptide-1 secretion in diabetic rats. J Ethnopharmacol.

[10] Zhang Q, Ye YL, Yan YX, Zhang WP, Chu LS, Wei EQ (2009). Protective effects of Huanglian-Jie-Du-Tang on chronic brain injury after focal cerebral ischemia in mice. Zhejiang Da Xue Xue Bao Yi Xue Ban.

[11] Xu J, Murakami Y, Matsumoto K, Tohda M, Watanabe H, Zhang S (2000). Protective effect of Oren-gedoku-to (Huang-Lian-Jie-Du-Tang) against impairment of learning and memory induced by transient cerebral ischemia in mice. J Ethnopharmacol.

[12] Hwang YS, Shin CY, Huh Y, Ryu JH (2002). Hwangryun-Hae-Dok-tang (Huanglian-Jie-Du-Tang) extract and its constituents reduce ischemia-reperfusion brain injury and neutrophil infiltration in rats. Life Sci.

[13] Ohta Y, Kongo-Nishimura M, Hayashi T, Kishikawa T (2004). Effect of Oren-gedoku-to (Huanglian-Jie-Du-Tang) extract on disruption of hepatic antioxidant defense systems in rats treated with D-galactosamine. J Ethnopharmacol.

[14] Hsu YL, Kuo PL, Tzeng TF, Sung SC, Yen MH, Lin LT (2008). Huang-lian-jie-du-tang, a traditional Chinese medicine prescription, induces cell-cycle arrest and apoptosis in human liver cancer cells *in vitro* and *in vivo*. J Gastroenterol Hepatol.

[15] Sekiya N, Kainuma M, Hikiami H, Nakagawa T, Kouta K, Shibahara N (2005). Oren-gedoku-to and Keishi-bukuryo-gan-ryo inhibit the progression of atherosclerosis in diet-induced hypercholesterolemic rabbits. Biol Pharm Bull.

[16] Ross R (1993). The pathogenesis of atherosclerosis: a perspective for the 1990s. Nature.

[17] Ross R (1999). Atherosclerosis is an inflammatory disease. Am Heart J.

[18] Re R, Pellegrini N, Proteggente A, Pannala A, Yang M, Rice-Evans C (1999). Antioxidant activity applying an improved ABTS radical cation decolorization assay. Free Radical Biol Med.

[19] Moreno MI, Isla MI, Sampietro AR, Vattuone MA (2000). Comparison of the free radical-scavenging activity of propolis from several regions of Argentina. J Ethnophamacol.

[20] Barnhart RL, Busch SJ, Jackson RL (1989). Concentration-dependent antioxidant activity of probucol in low density lipoproteins *in vitro*: probucol degradation precedes lipoprotein oxidation. J Lipid Res.

[21] Buege JA, Aust SD (1978). Microsomal lipid peroxidation. Methods Enzymol.

[22] Sparks DL, Phillips MC (1992). Quantitative measurement of lipoprotein surface charge by agarose gel electrophoresis. J Lipid Res.

[23] Janero DR (1990). Malondialdehyde and thiobarbituric acid-reactivity as diagnostic indices of lipid peroxidation and peroxidative tissue injury. Free Radic Biol Med.

